# Isolation, characterization and phylogenetic analyses of avian influenza A (H9N2) viruses isolated from poultry between 2019 and 2023 in Egypt

**DOI:** 10.1186/s12917-025-04514-4

**Published:** 2025-07-11

**Authors:** Zienab Mosaad, Naglaa M. Hagag, Moataz M. Elsayed, Wesam H. Mady, Ali M. Zanaty, Zeinab A. El-Badiea, Fatma Amer, Mahmoud Said, Abdullah Selim, Eman Farghally, Samah Eid, Amany Adel, Mahmoud M. Naguib, Mohamed E. El Zowalaty, Momtaz A. Shahein

**Affiliations:** 1 Reference Laboratory for Veterinary Quality Control On Poultry Production, Animal Health Research Institute, Agriculture Research Center, Giza, Egypt; 2 Department of Medical Biochemistry and Microbiology, Uppsala University, Uppsala, Sweden; 3 Department of Microbiology and Immunology, Faculty of Pharmacy, Ahram Canadian University, Giza, Egypt

**Keywords:** Avian influenza, Poultry, Real-time RT-PCR, H9N2, Hemagglutinin, Neuraminidase, Live bird markets, Genome sequence, Egypt

## Abstract

The current study aimed to investigate the genetic characterization and evolution of low pathogenic avian influenza virus H9N2 in Egypt. Ten H9N2 viruses were recently isolated from samples collected between 2019 and 2023. Phylogenetic analysis of the haemagglutinin (HA) gene segment of the H9N2 isolates showed a relatedness with G1 H9 4.2 lineage and clustered within genotype III of the Egyptian strains identified earlier in 2018. The majority of H9N2 strains had seven and eight glycosylation sites in HA and neuraminidase (NA) respectively. All strains carried H191 and L234 residues in their hemagglutinin which are markers facilitating avian-to-human cross species barrier transmission. No stalk deletions were detected in NA gene. In addition, genetic analysis of the NA and M encoding proteins revealed the absence of substitutions associated with resistance to oseltamivir and amantadine. The NA showed S372A and R403W substitutions which were previously detected in H3N2 and H1N2 viruses that were reported in previous influenza pandemics in 1975 and 2001 respectively. Many mutations associated with virulence and mammalian infection were detected in internal proteins such as PB2(V504), PB1-F2(N66), PA (V127, L672, and L550), M2(S64), and NS1(42S). Analysis showed the presence of full-length PB1-F2 with ^227^PDZ^230^ motif which is associated with virus virulence and pathogenesis. Mammalian associated mutations such as PB2 (I 667, T64), PB1-P13, PB1-F2-S82, NP-K214, NP-Q398 and M1-I15 were detected. The HA gene was under positive selection pressure especially at sites 198 and 235 of RBS, while other internal genes were under negative selection pressure. The study highlights the importance of continuous monitoring of H9N2 virus to enable timely implementation of control measures in poultry populations in Egypt.

## Introduction

Influenza A viruses belonging to the *Orthomyxoviridae* family are naturally circulating in wild bird reservoir and continue to spread and cause infections and outbreaks in other species including domestic birds, and mammals including humans. Avian influenza viruses (AIVs) continue to pose significant morbidity and mortality globally and are the primary source of acute respiratory tract infections in poultry. Based on pathogenicity, AIVs can be categorized into two groups based on their disease outcomes into low-pathogenic avian influenza viruses (LPAIVs) which cause predominantly respiratory sickness and highly-pathogenic avian influenza viruses (HPAIVs) which can cause up to 100% mortality [1, 2]. Influenza A viruses are characterized by enveloped, pleomorphic virion particles with eight negative-sense RNA genome segments [3].

Low-pathogenic avian influenza virus H9N2 subtype is frequently detected in wild birds worldwide and is extensively present in poultry across different parts of Africa and Eurasia [3]. The occasional cases of human infections that have been reported since 1998 in China, Hong Kong, Bangladesh, Egypt, Pakistan, and Oman pose a concern to public health [3–7]. In Wisconsin, USA, LPAIV H9N2 was initially recorded in turkeys in 1966 [8]. The LPAIV H9N2 infection is usually associated with low morbidity and mortality, however co-infection with bacteria such as *Escherichia coli* and *Mycoplasma gallisepticum*, or with other viruses such as Newcastle Disease virus (NDV) and Infectious Bronchitis virus (IBV), can worsen infections and result in significant higher mortality rates [9]. Several studies reported that H9N2 virus infection may not cause clinical symptoms [10–12], albeit, additional studies are required.

Despite being reported as a low pathogenicity type, the viral genome of H9N2 subtype encodes internal genes that could result in the emergence of reassortant viruses and result in emerging viruses of pandemic threats [3, 13, 14]. The internal gene segments of the LPAIV H9N2 has contributed to the emergence of the HPAIV goose/Guangdong H5N1 virus in 1997, and LPAIV H7N9 viruses in 2013 [15, 16]. Additionally, internal genes from H9N2 viruses were acquired by human-adapted AIV strains such H5N6, H10N8, and H3N8 viruses, and these reassortant viruses can directly infect humans [17].

The LPAIV H9N2 embraces two phylogenetic lineages; the North American lineage which commonly circulates in wild birds and the Eurasian lineage which is divided into three lineages with different prototypes: A/quail/Hong Kong/G1/1997 (G1 lineage), A/chicken/ Beijing/1/94 and A/duck/HK-Y280–1997 (Y280 lineage), and A/chicken/Hong Kong/Y439/1997 (Korean lineage) [18–20]. The G1 lineage is the most prevalent globally circulating viruses, with four genetically classified groups (A, B, C, and D) [21].

In Egypt, the first record of the LPAIV H9N2 was from bobwhite quail in 2011 [22]. Since then, the virus circulated continuously in poultry populations and became endemic. Additionally, H9N2 was reported to cause sporadic human infections with several reported cases. The LPAIVs H9N2 isolated from Egypt are closely related to the H9N2 viruses reported from other regions which belonged to the Asian G1-like viruses [23, 24]. The poultry sector has suffered significant losses as a result of the ongoing outbreak of LPAI H9N2 viruses in Egyptian farms over the last ten years [25–27]. The virus persisted in its evolution, and in 2014 the quail/2014 variant a novel, antigenically different variation was found [28]. In the same year, natural reassortment of wild bird-like AIVs (PB2, PB1, PA, NP, NS) and an Egyptian H9N2/2011 virus (HA, NA, M) led to the detection of a novel LPAI H9N2 virus variant in pigeons in Egypt [29]. The pigeon H9N2 virus then experienced a second spontaneous reassortment event in 2014–2015, sharing just PB2, PB1, PA, and NS with an Egyptian virus in late 2014 [30, 31].

In addition to H9N2 subtype, HPAIVs H5N1 and H5N8 subtypes are circulating among poultry population in Egypt [32]. The continuous co-circulation of H9N2 with other subtypes increases the potential for reassortment events and the emergence of novel influenza strains with unknown pathogenicity and transmissibility [30, 33–35]. It was previously reported that chickens and pigeons were infected with reassorted H9N2 strains which were derived from various influenza subtypes originally found in wild birds [29, 36, 37]. Therefore, poultry faces real threats due to H9 viruses despite their low pathogenicity nature [38, 39]. The co-circulation of H5N1 and the potentially zoonotic LPAI H9N2 viruses in poultry farms and live bird markets have increased the risk of human exposure, resulting in challenges in epidemiological control and raising grave concerns for the emergence of new influenza A viruses of pandemic potential [40, 41].

The current study aimed to determine the genetic characterization of LPAIVs H9N2 isolated from poultry farms, live bird markets and backyard chicken in Egypt between 2019–2023 to monitor the genetic development of H9N2 viruses in Egypt and to explore the probability of the presence of novel reassortment(s).

## Material and method

### Sample collection

Between 2019 and 2023, the Reference Laboratory for Veterinary Quality Control on Poultry Production (RLQP) collected a total of 13240 samples for periodic surveillance effort for the presence of avian viruses. Cloacal and oropharyngeal swabs were collected from household, farm (live bird market), abattoir, and checkpoint sectors and included several kinds of poultry species. The samples were collected prior to slaughter from 19 governorates (Al Monofiya, Al Giza, Al Behera, Al Daqahlia, Benisueif, Al Gharbia, Al Fayioum, Al Minia, Al Qalyubia, Al Wadi Algidid, Ismaliya, Qena, Asyut, Al Sharqia, Alexandria, Damietta, Aswan, South Sinai, and Kafr Alsheikh). The swab samples were collected as previously published [42, 43].

### Virus detection using real time RT-PCR

Viral RNA was extracted from the poultry samples using QIAamp viral RNA Mini kit (Qiagen, Hilden, Germany) according to the manufacturer's instructions. In brief, RNA extracts were tested for the presence of influenza A virus matrix (M) gene as previously reported [44, 45]. Samples that were Influenza A virus positive by matrix-gene specific primers (cycle threshold (C_t_) was ≤ 14 or less) were further screened for the presence of H5, H7 and H9 viruses using H5, H7 and H9 subtype-specific primers and TaqMan probes using one-step real-time RT-PCR as previously reported [26, 46]. All samples were additionally screened for the presence of possible co-infection with other avian viruses including infectious bronchitis virus [47] and Newcastle disease virus [48] using RT-qPCR Verso one-step™ Real Time PCR kit (Thermo Scientific, Catalogue number AB4101A) using Stratagene MX3005 real-time PCR equipment as previously reported [43]. RNA template of known AIV subtypes was used as positive control with cycle threshold (*C*_t_) < 35. Nuclease-free water was used as the template for negative control.

### Influenza virus isolation

Influenza A virus RT-qPCR-positive samples were cultured for live virus isolation by inoculation in SPF embryonated chicken eggs (ECEs) as previously reported [49]. A volume of 100 μl from each swab sample was inoculated via the allantoic route of three 10-day-old ECEs per sample and incubated at 37°C for 72 h. The allantoic fluid was collected and tested for hemagglutination using 0.5% chicken erythrocytes as previously reported [49].

### Influenza virus whole genome sequencing

The HA, NA, M, PA, PB1, PB2, NP and NS genes of AIV-H9N2 positive samples were amplified using specific primers as previously reported [26, 50]. Amplifications were performed using Applied Biosystem Thermal Cycler (ProFlexTM PCR System) and Easyscript One-Step RT-PCR Kit (Trans Gen Biotech) according to manufacturer’s instructions. The PCR products were extracted by gel electrophoresis and purified using the QIAquick Gel Extraction Kit (Qiagen, Hilden, Germany). Amplicons were sequenced by the ABI PRISM® 3500 Genetic Analyzer (Life Technologies, USA) at the Genome Unit (Animal Health Research Institute, Agriculture Research Center) using the Big Dye Terminator v3.1 Cycle Sequencing Kit (Applied Biosystems, Waltham, MA, USA) and the Thermo Fisher Centrisep spin columns were used to further purify the products.

### Sequencing and phylogenetic analysis

Multiple sequence analysis and the generation of consensus sequences were performed as previously reported [51] using the Bio-edit software version 7.2.5 [52]. Using BioEdit nucleotide sequences were aligned against the H9N2 vaccine strains (accession numbers JQ440373.2 and JQ419502) currently deployed in Egypt and additional AIV strains representing distinct clades, were retrieved from the National Centre for Biotechnology Information (NCBI). Additional sequences were retrieved from the NCBI as of 22 February 2024 and phylogenetic analysis was performed using MEGA 6 [53]. The General Time Reversible (GTR) substitution with a moderate strength neighbor-joining technique, 1000 bootstrap repeats and an estimate of the proportion of invariable sites (I) were the best models [53]. Bio-edit software version 7.2.5 was used to calculate the pairwise nucleotide percentage identity [52]. Additionally, NetNGlyc 1.0 Server (http://www.cbs.dtu.dk/services/NetNGlyc/) was used to analyze the N-linked glycosylation pattern of the HA gene of the H9N2 viruses tested in the present study.

### Selective pressure and receptor binding key site analyses

The complete coding sequences from the ten H9N2 isolates were used to estimate the rate of non-synonymous and synonymous mutations at each site using Single-likelihood ancestor counting (SLAC) and MEME using the Datamonkey online version of HyPhy package.6 [54], using a significance threshold of ≤ 0.05 for both SLAC and MEME. Only locations identified by both approaches were considered positively selected [55]. All the sequences in every branch were analyzed by ratio estimation of non-synonymous (dN) to synonymous (dS) substitutions (ω = dN/dS) on a codon-by-codon basis, and ω < 1 indicates negative or purifying selective pressure; ω = 1 implies neutral evolution; and ω > 1 shows positive selection. BioEdit and MEGA 6.0 tools [53] were used for the analysis of the sequence format conversion and key amino acid changes and to analyze sequence homologies [56]. The prediction of potential N-glycosylation sites was performed using the NetNGlyc server 1.0.9 (http://www.cbs.dtu.dk/services/NetNGlyc).

## Results

### Phylogenetic analysis of Influenza A H9N2 viruses

**Fig. 1:**
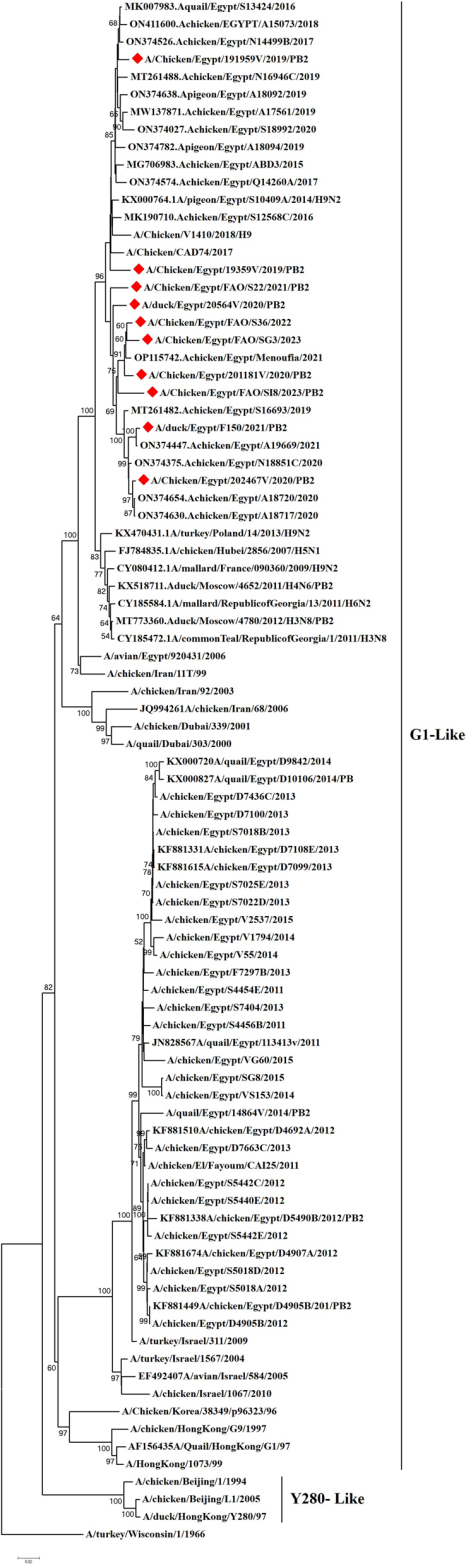
Phylogenetic tree of the PB2 gene of avian influenza subtype H9N2 viruses isolated in Egypt during 2020–2023 and reference isolates retrieved from GenBank. Phylogenetic analysis was conducted by using the neighbor-joining algorithm with the Kimura 2-parameter model. Strain A/turkey/Wisconsin/1/1966 was used as the root for the tree and the reliability of the phylogenetic tree inference at each node was estimated by the bootstrap method with 1,000 replications. Evolutionary analysis was conducted using MEGA6. A red rhomboid indicates isolates which were sequenced in the present study

**Fig. 2:**
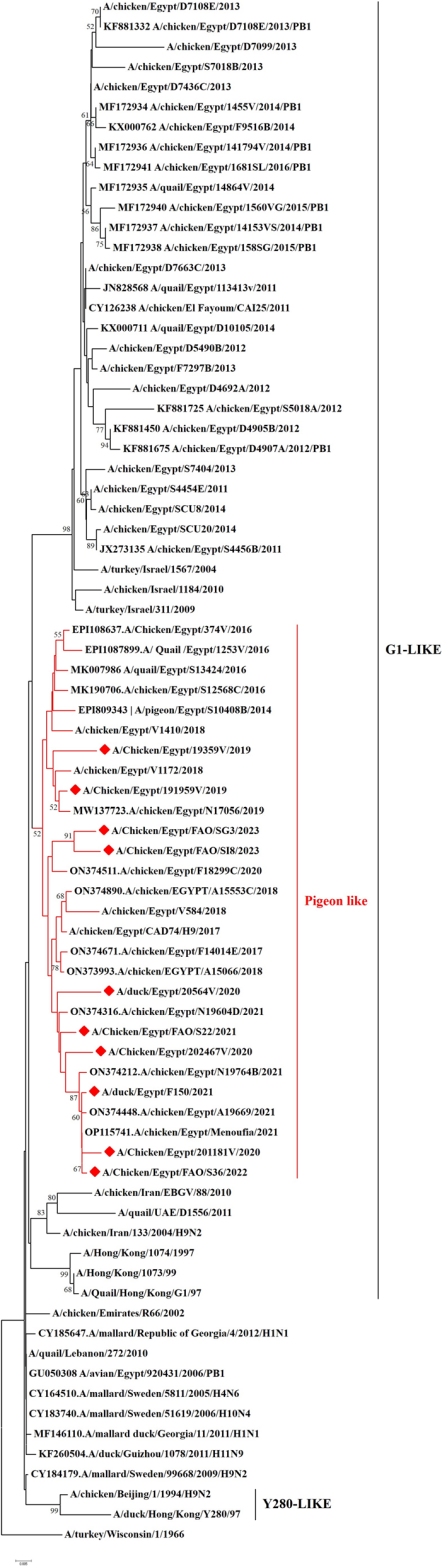
Phylogenetic tree of the PB1 gene of avian influenza subtype H9N2 viruses isolated in Egypt during 2020–2023 and reference isolates retrieved from GenBank. Phylogenetic analysis was conducted by using the neighbor-joining algorithm with the Kimura 2-parameter model. Strain A/turkey/Wisconsin/1/1966 was used as the root for the tree and the reliability of the phylogenetic tree inference at each node was estimated by the bootstrap method with 1,000 replications. Evolutionary analysis was conducted by using MEGA6. A red rhomboid indicates isolates which were sequenced in the present study

**Fig. 3:**
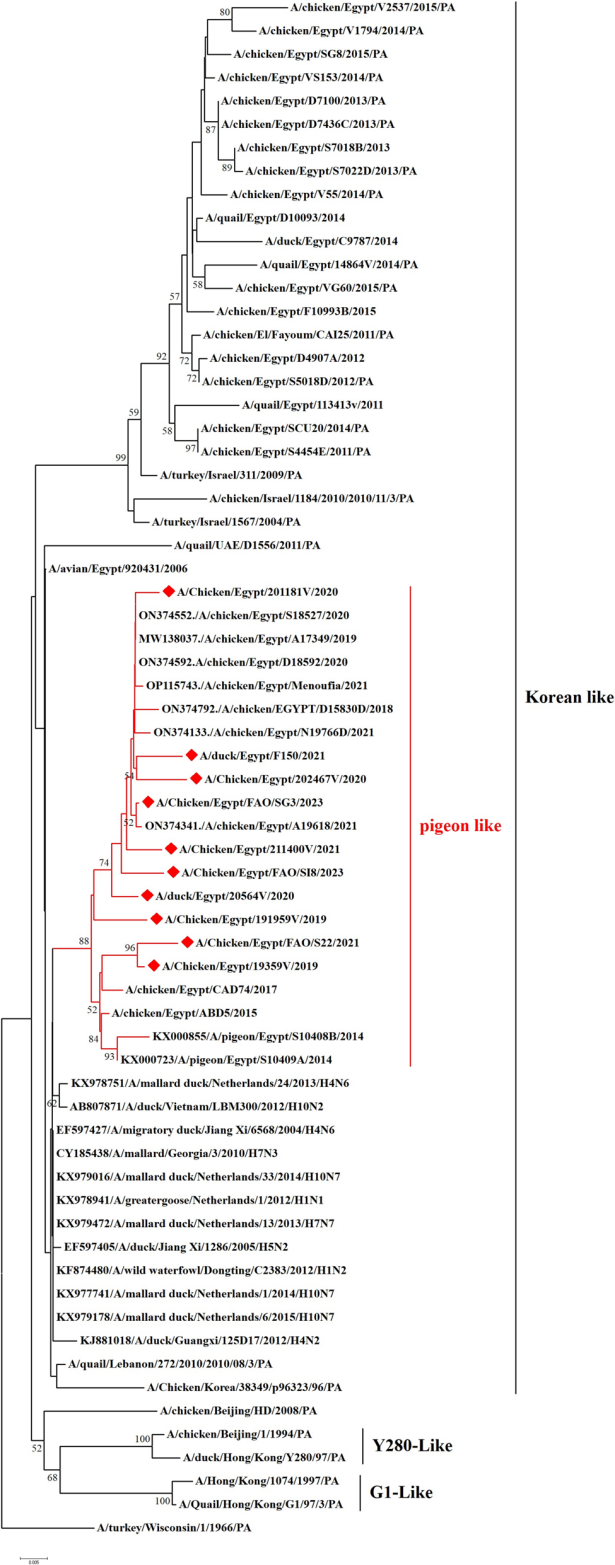
Phylogenetic tree of the PA gene of avian influenza subtype H9N2 viruses isolated in Egypt during 2020–2023 and reference isolates retrieved from GenBank. Phylogenetic analysis was conducted by using the neighbor-joining algorithm with the Kimura 2-parameter model. Strain A/turkey/Wisconsin/1/1966 was used as the root for the tree and the reliability of the phylogenetic tree inference at each node was estimated by the bootstrap method with 1,000 replications. Evolutionary analysis was conducted by using MEGA6. A red rhomboid indicates isolates which were sequenced in the present study

**Fig. 4:**
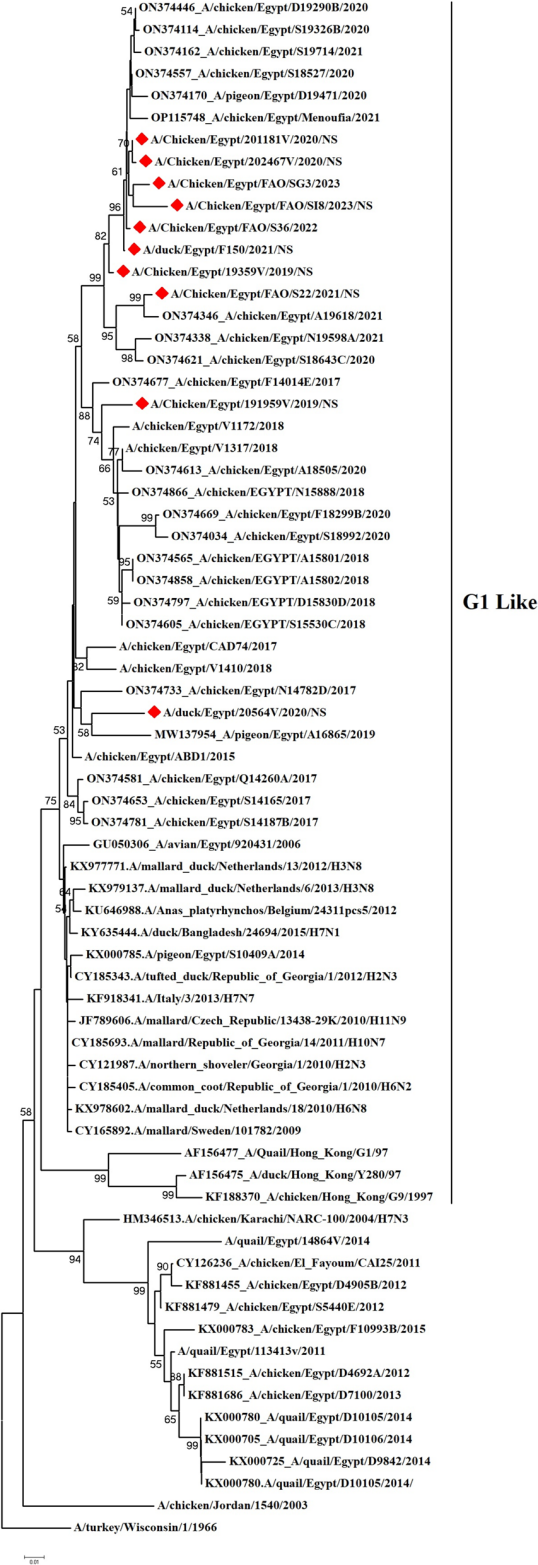
Phylogenetic tree of the NS gene of avian influenza subtype H9N2 viruses isolated in Egypt during 2020–2023 and reference isolates retrieved from GenBank. Phylogenetic analysis was conducted by using the neighbor-joining algorithm with the Kimura 2-parameter model. Strain A/turkey/Wisconsin/1/1966 was used as the root for the tree and the reliability of the phylogenetic tree inference at each node was estimated by the bootstrap method with 1,000 replications. Evolutionary analysis was conducted by using MEGA6. A red rhomboid indicates isolates which were sequenced in the present study

**Fig. 5:**
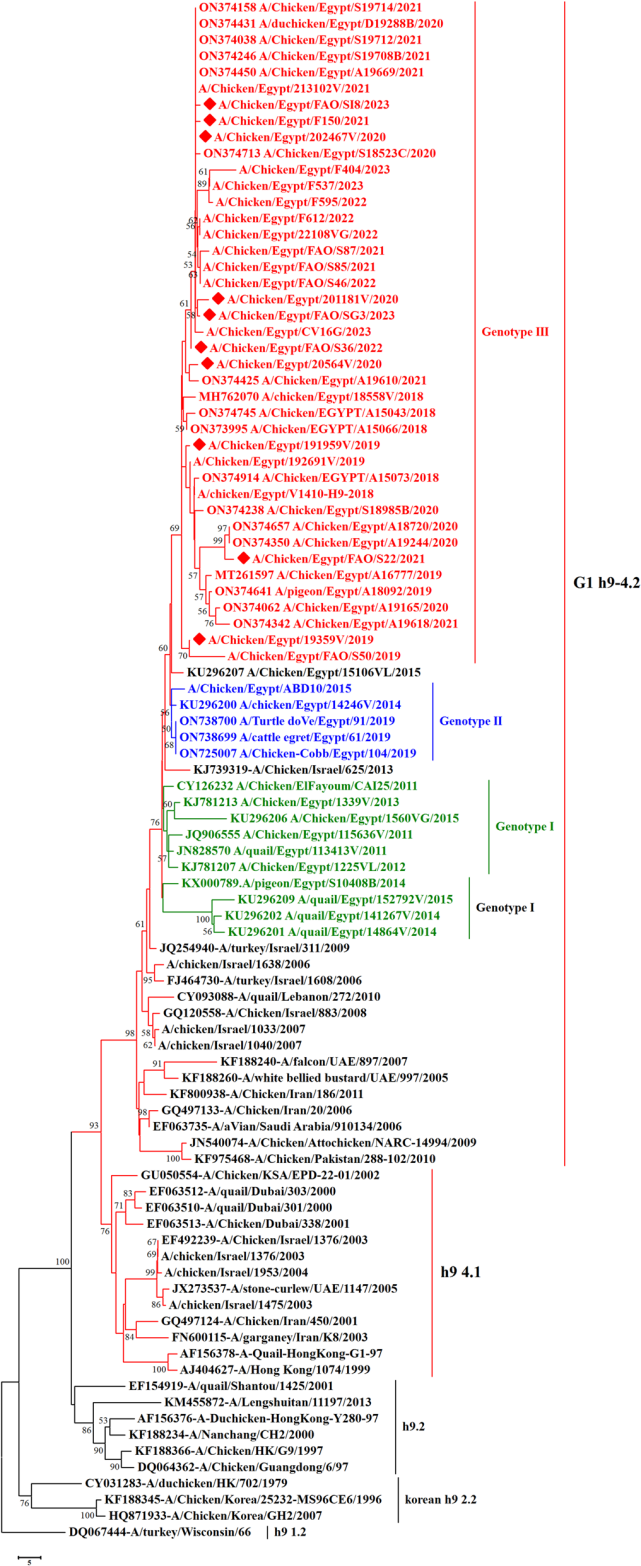
Phylogenetic tree of the HA gene of avian influenza subtype H9N2 viruses isolated in Egypt during 2020–2023 and reference isolates retrieved from GenBank. Phylogenetic analysis was conducted by using the neighbor-joining algorithm with the Kimura 2-parameter model. Strain A/turkey/Wisconsin/1/1966 was used as the root for the tree and the reliability of the phylogenetic tree inference at each node was estimated by the bootstrap method with 1,000 replications. Evolutionary analysis was conducted by using MEGA6. A red rhomboid indicates isolates which were sequenced in the present study

**Fig. 6:**
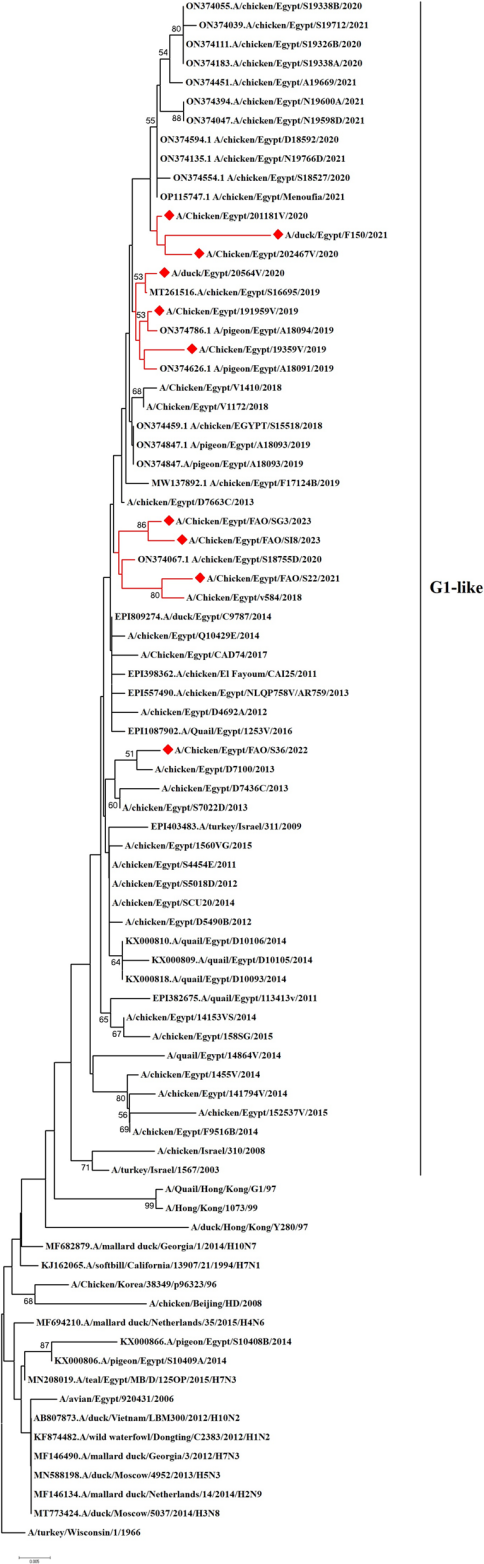
Phylogenetic tree of the NP gene of avian influenza subtype H9N2 viruses isolated in Egypt during 2020–2023 and reference isolates retrieved from GenBank. Phylogenetic analysis was conducted by using the neighbor-joining algorithm with the Kimura 2-parameter model. Strain A/turkey/Wisconsin/1/1966 was used as the root for the tree and the reliability of the phylogenetic tree inference at each node was estimated by the bootstrap method with 1,000 replications. Evolutionary analysis was conducted by using MEGA6. A red rhomboid indicates isolates which were sequenced in the present study

**Fig. 7:**
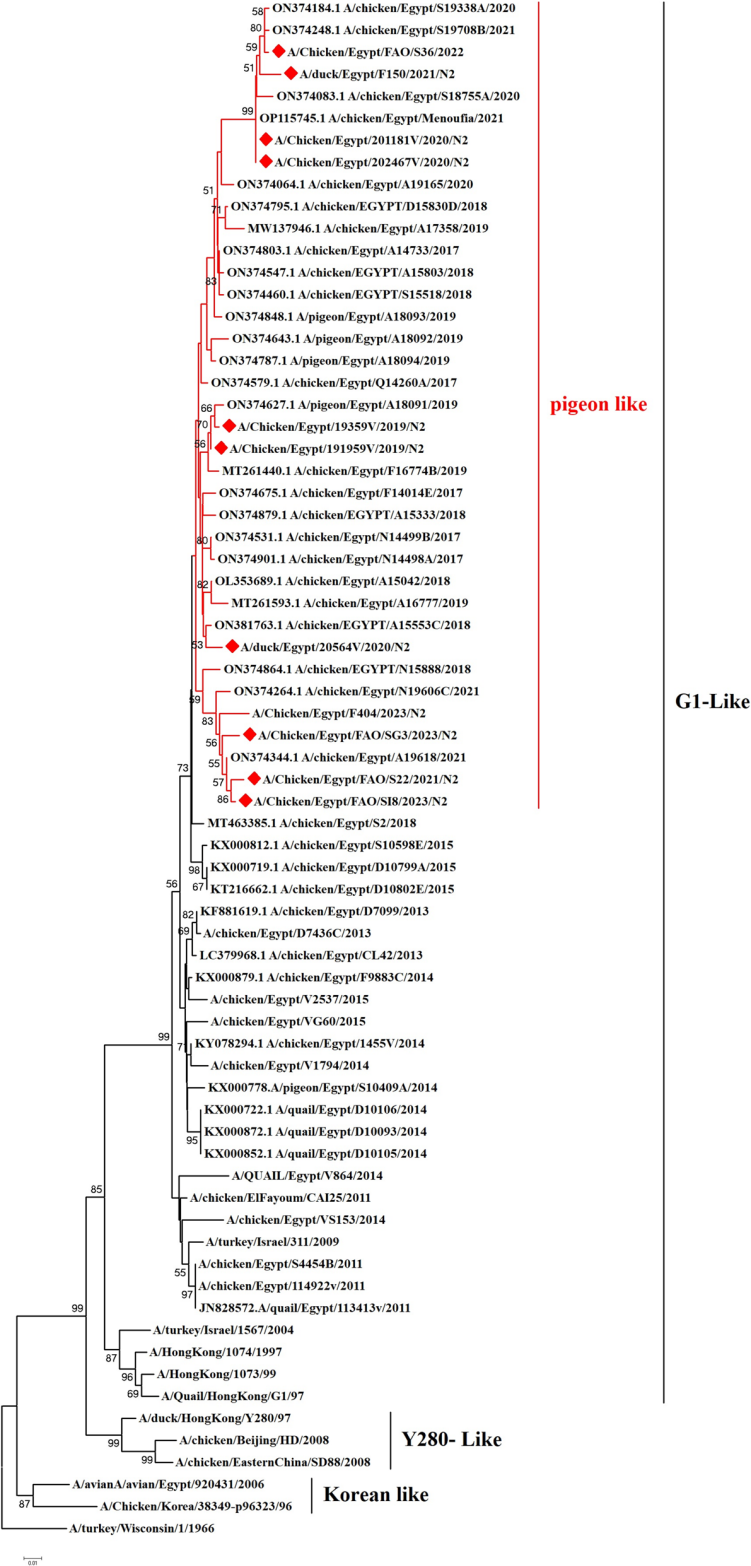
Phylogenetic tree of the NA gene of avian influenza subtype H9N2 viruses isolated in Egypt during 2020–2023 and reference isolates retrieved from GenBank. Phylogenetic analysis was conducted by using the neighbor-joining algorithm with the Kimura 2-parameter model. Strain A/turkey/Wisconsin/1/1966 was used as the root for the tree and the reliability of phylogenetic tree inference at each node was estimated by the bootstrap method with 1,000 replications. Evolutionary analysis was conducted by using MEGA6. A red rhomboid indicates isolates which were sequenced in the present study

**Fig. 8:**
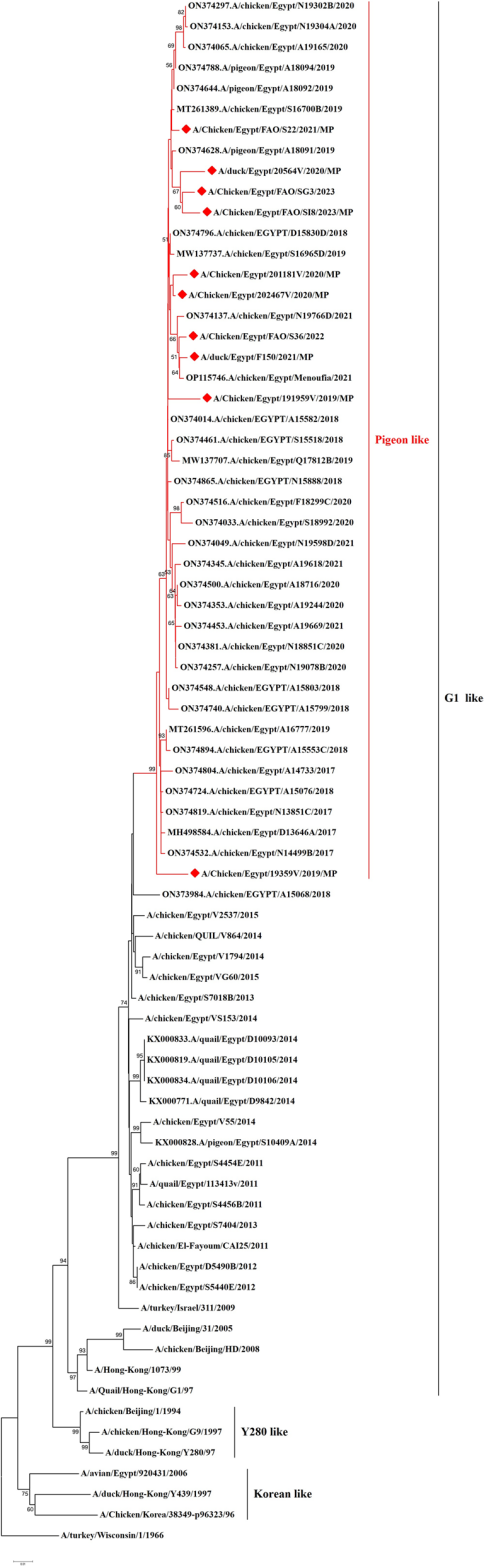
Phylogenetic tree of the MP gene of avian influenza subtype H9N2 viruses isolated in Egypt during 2020–2022 and reference isolates retrieved from GenBank. Phylogenetic analysis was conducted by using the neighbor-joining algorithm with the Kimura 2-parameter model. Strain A/turkey/Wisconsin/1/1966 was used as the root for the tree and the reliability of the phylogenetic tree inference at each node was estimated by the bootstrap method with 1,000 replications. Evolutionary analysis was conducted by using MEGA6. A red rhomboid indicates isolates which were sequenced in the present study

We have recorded a total of 332 positive cases of LPAI H9N2 outbreak during 2019 to 2023. All positive samples showed the absence of co-infection with other avian viruses such as infectious bronchitis virus and Newcastle disease virus using RT-qPCR. In the present study, ten H9N2 isolates were investigated for genetic characterization using whole viral genome sequencing. The selection of ten Influenza A viruses H9N2 subtype representing the common circulating strains during 2019–2023 as shown in (Table 1). Phylogenetic analysis of the ten Egyptian H9N2 isolates reported in the current study showed that the PB2 (Fig. 1), PB1 (Fig. 2), PA (Fig. 3), and NS (Fig. 4) genes have high identity to H9N2 viruses isolated previously from domestic pigeons in Egypt in 2014 (accession number KX000789), while HA (Fig. 5), NP (Fig. 6), NA (Fig. 7), and MP (Fig. 8) grouped with the previous Egyptian H9N2 isolates from 2015–2021 showed high identity to H9N2 viruses isolated previously from Egypt grouped in the prototype A/quail/Hong Kong/G1/97 (G1-like) of the Eurasian lineage rather than other AIV H9N2 prototypes such as A/duck/Hong Kong/Y280/97 and A/chicken/Hong Kong/G9/1997. All Egyptian strains investigated in the present study were also related to each other with an identity ranging from 98 to 100% for the HA. The strains were also monophyletic with recently reported Egyptian AIV H9N2 sequences during 2021 with high nucleotide sequence identities 97–99%. The HA gene of the current H9N2 isolates was found to be related to G1 H9-4.2 lineage clustered with genotype III.

**Table 1:** Epidemiological data and GenBank accession numbers of the eight segments of influenza H9N2 viruses isolated from oropharyngeal swab samples obtained from poultry species in Egypt between 2019 and 2023 in the present study

**AIV isolate ID**	**Governorate**	**Host location**	**Date of collection**	C_t_ **value**	**PB2**	**PB1**	**PA**	**HA**	**NP**	**NA**	**MP**	**NS**
A/chicken/Egypt/191959 V/2019	Al Giza	Farm	April, 2019	12	PP345561	PP345544	PP345535	PP345490	PP345524	PP345513	PP345918	PP346094
A/chicken/Egypt/19359 V/2019	Al Gharbia	Backyard	January, 2019	11	PP345560	PP345545	PP345534	PP345489	PP345525	PP345514	PP345919	PP346095
A/chicken/Egypt/202467 V/2020	Al Menoufia	Farm	July, 2020	14	PP345563	PP345547	PP345538	PP345492	PP345527	PP345516	PP345920	PP346097
A/duck/Egypt/20564 V/2020	Asyut	Farm	February, 2020	11	PP345562	PP345546	PP345536	PP345491	PP345526	PP345515	PP345921	PP346096
A/chicken/Egypt/201181 V/2020	Al Qalyubia	Farm	March, 2020	9	PP345564	PP345548	PP345537	PP345493	PP345528	PP345517	PP345922	PP346098
A/duck/Egypt/F150/2021	Al Behera	Farm	February, 2021	12	PP345565	PP345549	PP345539	PP345494	PP345529	PP345518	PP345923	PP346099
A/chicken/Egypt/FAO/S22/2021	Al Minia	Live bird market	May, 2021	11	PP345566	PP345550	PP345540	PP345495	PP345530	PP345519	PP345924	PP346100
A/chicken/Egypt/FAO/S36/2022	Al Qalyubia	Live bird market	April, 2022	13	PP345567	PP345551	PP345541	PP345496	PP345531	PP345520	PP345925	PP346101
A/chicken/Egypt/FAO/SI8/2023	Beni Suef	Live bird market	June, 2023	14	PP345569	PP345553	PP345542	PP345498	PP345533	PP345522	PP345926	PP346103
A/chicken/Egypt/FAO/SG3/2023	Port Said	Live bird market	January, 2023	8	PP345568	PP345552	PP345543	PP345497	PP345532	PP345521	PP345927	PP346102

### Genetic analysis of influenza A H9N2 viruses

#### Surface glycoproteins

The existence of multiple amino acid changes at the antigenic epitope sites of the HA was identified by genetic investigation of several antigenic sites in the HA gene of H9N2 viruses. Based on the genetic analysis of the HA cleavage motif, all the H9N2 strains in the present study showed RSSR*GLF (Table 2). Several receptor binding sites (RBS) of the HA of H9N2 viruses were examined which are the driving variables related with the capacity of the virus to attach to mammalian host cellular receptors such as H191, T197, N232, L234, I235, and G236 (H9 numbering). All the tested H9N2 isolates included N166, as previously reported in A/quail/Hong Kong/G1/97 (Table 2). On the contrary, alanine (A) was detected at position 198 (H9 numbering) in all the H9N2 isolates in the present study, except for A/chicken/Egypt/201181V/2020 which had thymidine (T) at the same position as previously detected in A/quail/Hong Kong/Y280/97 (H9N2) as shown in Table 2.

**Table 2:** Comparison of amino acid sequences of the HA of H9N2 viruses isolated from poultry in Egypt between 2019 and 2023 with ancestor H9N2 viruses (H9 numbering) at receptor binding site (RBS), cleavage site and glycosylation sites

**No**	**Strain ID**	**Receptor binding site**								**Glycosylation site**										
		**166**	**191**	**197**	**198**	**232**	**234**	**235**	**236**	**Cleavage site**	**29**	**105**	**141**	**206**	**218**	**258**	**298**	**305**	**492**	**551**
**1**	A/quail/Hong Kong/G1/97	S	H	T	E	N	L	Q	G	**RSSR/GLF**	**NSTE**	**NGTC**	**NVTY**	**NDTT**	**NRTF**	**NGTS**	**NSTL**	**NISK**	**NGTY**	**NGSC**
**2**	A/duck/Hong Kong/Y280/97	N	N	-	T	-	-	-	-	**RSSR/GLF**	----	X	----	X	----	X	----	----	----	----
**3**	A/turkey/Wisconsin/1/1966	-	-	**-**	-	-	Q	-	-	**VSSR/GLF**	----	X	----	X	----	X	----	----	----	----
**4**	A/quail/Egypt/14864 V/2014	N	-	-	V	-	Q	I	-	**RSSR/GLF**	----	X	----	**NTT**	X	X	----	----	----	----
**5**	A/quail/Egypt/113413v/2011	**N**	-	-	**A**	-	-	**I**	-	**RSSR/GLF**	----	----	----	X	X	X	----	----	----	----
**6**	A/pigeon/Egypt/S10409A/2014	**N**	-	-	**A**	-	-	-	-	**RSSR/GLF**	----	----	----	X	X	X	----	----	----	----
**7**	**A/chicken/Egypt/FAO/SG3/2023**	**N**	-	-	**A**	-	-	-	-	**RSSR/GLF**	----	----	----	X	X	X	----	----	----	----
**8**	**A/chicken/Egypt/FAO/SI8/2023**	**N**	-	-	**A**	-	-	**I**	-	**RSSR/GLF**	----	----	----	X	X	X	----	----	----	----
**9**	**A/chicken/Egypt/FAO/S36/2022**	**N**	-	-	**A**	-	-	**I**	-	**RSSR/GLF**	----	----	----	X	X	X	----	----	----	----
**10**	**A/duck/Egypt/F150/2021**	**N**	-	-	**A**	-	-	**I**	-	**RSSR/GLF**	----	----	----	X	X	X	----	----	----	----
**11**	**A/chicken/Egypt/FAO/S22/2021**	**N**	-	-	**A**	-	-	**I**	-	**RSSR/GLF**	----	----	----	X	X	X	----	----	----	----
**12**	**A/duck/Egypt/20564 V/2020**	**N**	-	-	**A**	-	-	**I**	-	**RSSR/GLF**	----	----	----	X	X	X	----	----	----	----
**13**	**A/chicken/Egypt/201181 V/2020**	**N**	-	-	**T**	-	-	**I**	-	**RSSR/GLF**	----	----	----	X	X	X	----	----	----	----
**14**	**A/chicken/Egypt/202467 V/2020**	**N**	-	-	**A**	-	-	**I**	-	**RSSR/GLF**	----	----	----	X	X	X	----	----	----	----
**15**	**A/chicken/Egypt/191959 V/2019**	**N**	-	-	**A**	-	-	**I**	-	**RSSR/GLF**	----	----	----	X	X	X	----	----	----	----
**16**	**A/chicken/Egypt/19359 V/2019**	**N**	-	-	**A**	-	-	**I**	-	**RSSR/GLF**	----	----	----	X	X	X	----	----	----	----

The “-” refers to similar to A/quail/Hong Kong/G1/97 and “X” refers to absence of the glycosylation site

Isolates in the present study are shown in bold text

The Egyptian H9N2 viruses in the present study had seven N-linked glycosylation sites (^29^NSTE, ^105^NGTC, ^141^NVTY, ^298^NSTL, ^305^NISK, ^492^NGTYand^551^NGSC (H9 numbering), and all the ten isolates lacked two glycosylation sites at position 206 and 218 as compared to G1-like viruses (Table 2).

Genetic analysis of NA sequences showed that stalk deletion was not detected in any of the tested viruses in the present study. Analysis of the binding-pocket residues in the NA genes which are involved in interactions with antiviral drugs showed that the Egyptian H9N2 viruses had 119E, 198D, 222I, 274H, and 292R residues. Egyptian H9N2 viruses had NA genes with eight N-linked glycosylation sites (^44^ NTST,^61^ NITE,^69^ NGTI,^86^ NWSK,^146^ NGTT,^200^ NATA, 2^34^ NGTC,^402^ NRSG), the Egyptian strain A/chicken/Egypt/19359V/2019 had additional two glycosylation sites at (^329^ NDSS,^143^ NHSN), while the strain A/duck/Egypt/F150/2021 lack the glycosylation site (^44^ NTST). Genetic analysis of the hemadsorption sites (366–373,399–403, and 431–433), which are located on the surface of the NA, distant from the neuraminidase enzyme active site, revealed the presence of the ^366^IKKDSRAG^373^ motif in most of isolates while ^366^IK**T**DSRAG^373^ motif was detected in three strains in the current study (A/chicken/Egypt/FAO/SG3/2023, A/chicken/Egypt/FAO/SI8/2023and A/chicken/Egypt/FAO/S22/2021), the presence of the ^399^DSNNWS^404^ in most of isolates while ^399^DSNNRS^404^ was detected in (A/chicken/Egypt/FAO/SG3/2023, A/chicken/Egypt/FAO/SI8/2023 and A/chicken/Egypt/FAO/S22/2021), in addition to substitution (D^401^N) resulting in showing ^399^DSDNR^404^ in (A/chicken/Egypt/202467V/2020, A/Chicken/Egypt/191959V/2019 and A/chicken/Egypt/19359V/2019). The locus ^431^PQE^433^ was detected in most of isolates while, ^431^PHE^433^ was detected in (A/chicken/Egypt/FAO/S36/2022, A/duck/Egypt/F150/2021, A/chicken/Egypt/201181V/2020 and A/chicken/Egypt/202467V/2020). The neuraminidase revealed S^372^A and R^403^W alterations that were originally identified in H3N2 and H2N2 viruses which were previously reported in human pandemics.

### Internal proteins

Genetic analysis of PB2 segments of the ten Egyptian H9N2 isolates revealed the presence of mutations V504, D701N and E627K which were absent in all viruses analyzed (Table 1). The mammalian associated marker (V667I) was detected in strains A/chicken/Egypt/191959V/2019 and A/chicken/Egypt/19359V/2019. Moreover, mutation M64T associated with mammalian host specificity was detected in H9N2 strains in the present study isolated during 2019–2021. All H9N2 strains in the present study showed virulence substitution V504, however the mammalian associated substitutions D701N and E627K were absent in H9N2 the analyzed H9N2 strains. Other residues of PB2 were associated with avian preference as shown in (Table 1). PB1 exhibited proline (13P), in all other strains included in this study. All strains displayed a full-length (90 aa) PB1-F2 protein. The pathogenesis substitution of 66S PB1-F2, had been detected in A/chicken/Egypt/FAO/SG3/2023, the remaining strains in the present study show 66N. The mammalian preference substitution 82S was detected. Analysis showed that H9N2 viruses in the present study contained the three inflammatory amino acids 62L, 79R, and 82L. The PA gene of all Egyptian isolates had several genetic markers associated with virulence, including V127, L550, and L672 (Table 3) except for A/chicken/Egypt/FAO/S22/2021 which show 127I. In addition, mammalian host specificity substitutions 382D which detected in all strains, and R 57Q was detected in A/duck/Egypt/F150/2021 and A/chicken/Egypt/191959V/2019. Other residues of PA were associated with avian host-specific markers (Table 3).

**Table 3:** Analysis of virulence and host range genetic determinants in the viral internal proteins of H9N2 viruses isolated from poultry in Egypt between 2019 and 2023 with the ancestor H9N2 viruses in comparison to the reference strain

**No**	**Strain ID**	**PB2**			**PB1**	**PB1-F2**			**PA**						**NP**		**M1**	**M2**	**NS1**	**PDZ**	**DELETION 82–82**
		504	**64**	667	13	66	79	82	**57**	**127**	241	382	**550**	**672**	214	398	**15**	**64**	**42**	^**227**^**EPEV**^**230**^	**NO**
1	A/quail/Hong Kong/G1/97	V	M	I	P	N	R	L	R	V	C	E	L	L	R	Q	I	S	S	^**227**^**- - - -**^**230**^	NO
2	A/duck/Hong Kong/Y280/97	-	-	V	-	-	-	-	-	-	-	-	-	-	-	-	-	-	-	^**227**^**- S- - **^**230**^	**NO**
3	A/turkey/Wisconsin/1/1966	-	-	V	-	-	-	-	-	-	-	-	-	-	L	-	V	-	-	^**227**^**KS- - **^**230**^	NO
4	A/quail/Egypt/14864 V/2014	-	-	V	-	-	-	S	Q	-	-	-	-	-	K	-	-	F	-	^**227**^**KS- - **^**230**^	**NO**
5	A/quail/Egypt/113413v/2011	-	-	V	-	-	-	-	-	-	-	-	-	-	K	-	-	-	-	^**227**^**- S- - **^**230**^	NO
6	A/pigeon/Egypt/S10409A/2014	-	-	V	-	-	-	W	-	-	-	D	-	-	R	-	-	-	-	^**227**^**- S- - **^**230**^	**NO**
**7**	**A/chicken/Egypt/FAO/SG3/2023**	-	T	V	-	S	-	S	-	-	-	D	-	-	K	-	-	-	-	^**227**^**- S- - **^**230**^	NO
**8**	**A/chicken/Egypt/FAO/SI8/2023**	-	-	V	-	-	-	S	-	-	-	D	-	-	K	-	-	-	-	^**227**^**- S- - **^**230**^	**NO**
**9**	**A/chicken/Egypt/FAO/S36/2022**	-	T	V	-	-	-	S	-	-	-	D	-	-	K	-	-	-	-	^**227**^**- S- - **^**230**^	NO
**10**	**A/duck/Egypt/F150/2021**	-	T	V	-	-	-	S	Q	-	-	D	-	-	K	-	-	-	-	^**227**^**- S- - **^**230**^	NO
**11**	**A/chicken/Egypt/FAO/S22/2021**	-	-	V	-	-	-	S	-	I	-	D	-	-	K	-	-	-	-	^**227**^**- S- - **^**230**^	NO
**12**	**A/duck/Egypt/20564 V/2020**	-	T	V	-	-	-	S	-	-	-	D	-	-	K	-	-	-	-	^**227**^**- S- - **^**230**^	**NO**
**13**	**A/chicken/Egypt/201181 V/2020**	-	T	V	-	-	-	S	-	-	-	D	-	-	K	-	-	-	-	^**227**^**- S- - **^**230**^	NO
**14**	**A/chicken/Egypt/202467 V/2020**	-	T	V	-	-	-	S	-	-	-	D	-	-	K	-	-	-	-	^**227**^**- S- - **^**230**^	**NO**
**15**	**A/chicken/Egypt/191959 V/2019**	-	-	-	-	-	-	S	Q	-	-	D	-	-	K	-	-	-	-	^**227**^**- S- - **^**230**^	NO
**16**	**A/chicken/Egypt/19359 V/2019**	-	-	-	-	-	-	S	-	-	-	D	-	-	K	-	-	-	-	^**227**^**- S- - **^**230**^	**NO**

The “-” refers to similar to A/quail/Hong Kong/G1/97

Isolates in the present study are in bold text

The NP gene of Egyptian H9N2 isolates had amino acid substitutions associated with mammalian host specificity, such as 214K and 398Q. M1 protein showed the substitution 15I other host-specific markers of M1 were avian-like (Table 3). The M2 protein revealed the presence of virulence substitutions 64S, several amino acid substitutions associated with mammalian host specificity were observed in the M2 protein, such as the substitution of 16G, 28V and 55F was found at position in all viruses analyzed. All viruses analyzed revealed that the M2 protein had 26L, 27I, 30A, 31S, and 34G which is associated with the absence of resistance to amantadine. Genetic analysis of the NS1 protein of Egyptian isolates revealed that all viruses analyzed had 42S at position, which is a virulence marker (Table 3). The NS1 protein was 230 aa long and contained the ^227^ESEV^230^ PDZ ligand (PL) motif at the C-terminal end but the KSEV motif was not detected. The substitution of E for K/Rat position 227 in NS1, which is associated with mammalian host specificity has not been observed in any of the isolates. No deletions were detected at 80–84 in NS1. The NS2 protein of all the tested isolates had no residues associated with virulence at positions 31 and 56.

Selection pressure and molecular analyses have demonstrated that eight single amino acid polymorphism sites (58, 87, 198, 235, 239, 429, 430, and 539) of the HA gene were under positive selection pressure as shown in (Table 4), while other genes were under negative selection pressure. The most important substitutions at key receptor binding sites were 198 and 235.

**Table 4:** Positively selected sites in the hemagglutinin gene of Egyptian H9N2 viruses from different clusters using SLAC and MEME analysis

	**Codon number**	**S**	**N**	**dS**	**dN**	(ω**) Selection detected**	**Common amino acid**
**HA**	58	3.833	24.167	3.772	13.237	Pos. *p* = 0.010	K/R/M
	87	5.5	24.5	5.183	13.548	Pos. *p* = 0.025	M/I/T/S/L/V
	198	5.833	51.167	5.838	25.599	Pos. *p* = 0.000	E/A/T/V/S
	235	1	22	1.243	10.456	Pos. *p* = 0.006	I/Q/A/T
	239	3.417	16.583	3.438	8.611	Pos. *p* = 0.088	D/I/Y/N
	429	5	23	5.487	11.275	Pos. *p* = 0.095	I/V
	430	1.5	9.5	1.817	7.377	Pos. *p* = 0.083	W/G
	539	0.5	17.5	0.61	8.408	Pos. *p* = 0.012	T/I/M

dN = non-synonymous, dS = synonymous substitutions ω < 1 indicates negative or purifying selective pressure; ω = 1 implies neutral evolution; and ω > 1 shows positive selection

*P*-values of < 0.05 were significant

## Discussion

The continuous surveillance for LPAI- H9N2 in domestic poultry is critically required since the disease has huge impact on the poultry industry. Because LPAI- H9N2 is an endemic disease in Egypt, monitoring its prevalence in poultry farms is critical [57], due to the genetic evolution of the virus. Annual surveillance is performed routinely and LPAIV-H9N2 shows different patterns of spread. Furthermore, due to the co-circulation of other influenza subtypes in Egypt, LPAIV-H9N2 poses a risk of zoonotic transmission as was previously reported [40], however further investigations are required to delineate the zoonotic nature of these viruses, while no data were reported on human H9N2 infections in Egypt in recent years.

Based on the HA gene analysis, Egyptian H9N2 viruses have genetic clustered to G1 H9-4.2 (G1- subgroup B lineage) [21, 58], however LPAIV- H9N2 viruses reported from Egypt are constantly evolving [28, 40, 59]. In recent years, Egyptian H9N2 viruses have been grouped into three genotypes (I, II, and III) [31, 60]. Genotype I was found in Egypt between 2010 and 2013 [57, 61]. In 2014, genotype II came to light in wild pigeons (*Columba livia domestica*) carrying five segments (PB2, PB1, PA, NP, and NS) from Eurasian AIVs circulating in wild birds, as well as the HA, NA, and M genes from genotype I as previously reported [29]. Another reassortant form (genotype III) emerged between 2015 and 2016, containing four genes (PB2, PB1, PA, and NS) from genotype II and HA, NP, NA, and M genes from genotype I [60]. The findings of the current study demonstrate that the reassortant H9N2 virus of genotype III, continued to circulate in Egypt between 2019 and 2023 and genotypes strains have not been reported after 2014.

The hemagglutinin of Egyptian H9N2 viruses had K/RSSR*GLF cleavage motifs which is an evidence of the low pathogenicity nature of different H9N2 viruses isolated from the Middle East and Asia and the adaptation of these viruses to the chicken host [49, 62].

Several receptor binding sites (RBS) of the HA of H9N2 viruses were examined to determine variables related with the capacity of the virus to attach to mammalian host cellular receptors [63]. The Egyptian H9N2 strains reported in the current study had mammalian host substitutions, N191H and Q234L [64] and all H9N2 isolates included N166, as previously reported in A/quail/Hong Kong/G1/97 [65] (Table 2). The amino acid mutation A198 of the hemagglutinin reported in the H9N2 strains reported in the current study demonstrates the low affinity of hemagglutinin to human-like receptors than T198 which show higher affinity to α 2,6 human-like receptors as previously reported [21]. The Egyptian H9N2 viruses have seven N-linked glycosylation sites with conserved glycosylation sites at residue positions 298 (NSTL) and 305 (NISK) [59]. Compared to G1-like viruses, the H9N2 isolates in the present study lacked the glycosylation sites at positions 206 and 218 as was previously reported [61]*.* Analysis of NA genes of most strains in the present study revealed eight N-linked glycosylation sites, while specific strains had or lacked additional sites. It was suggested that increasing glycosylation of influenza viruses resulted in lower pathogenicity and virulence [66]. The HB site of the NA protein contains six amino acids which interact directly with sialic acids, including three residues at positions 367-372, 400 N, 403 W, and 432 K [67]. The two alterations (S372A and R403W) improved the cross-species barrier and adaptation to mammalian hosts. These alterations were also reported in H9N2 viruses recovered in Asia and the Middle East, particularly in the H2N2 and H3N2 subtypes which resulted in human influenza pandemics [68]. All Egyptian H9N2 strains in the present study were predicted to be sensitive to neuraminidase inhibitors based on their NA sequence analysis [69].

Mutations in the replication complex genes (PB2, PB1, and/or PA) may lead to increased viral replication [70]. The PA gene is reported to play a significant role in the virus adaptability to new hosts, and PA mutations may enhance virulence in animal models [71, 72]. The RNP complex also creates host-specific genetic lineages and markers. In addition, the M1 protein (M1) interacts with the viral RNP and may influence the host range [73].

Genetic analysis of PB2 segments of the ten Egyptian H9N2 isolates in the current study showed the presence of virulence marker V504 (Table 3) as previously reported [74]. Other mutations I667, and M64T which are reported to be associated with mammalian host specificity was detected [75]. PB1 showed proline (P13) along with all other strains included in this study. This amino acid serves as a marker for virulence in mice [73]. Analysis showed that all H9N2 strains had a full-length (90 aa) PB1-F2 protein that suggested increased pathogenicity of AIVs [76]. The substitution of 66S PB1-F2, which is associated with enhanced viral pathogenesis [77] was detected in strain A/Chicken/Egypt/FAO/SG3/2023, while the other H9N2 strains in the present study showed 66N. The ten H9N2 strains in the present study showed the 62L, 79R and 82L inflammatory amino acids [78], and the 82S mammalian preference substitution as previously reported [59]. The PA gene of all Egyptian H9N2 isolates had several genetic markers associated with virulence such as V127 and L550 and L672 [70]. Other residues of PA were associated with avian host-specific markers as shown in (Table 3). All the ten H9N2 viruses showed NP substitutions associated with mammalian host 214K and 398Q [79]. The M2 protein of Egyptian H9N2 isolates showed the presence of 64S amino acid substitutions associated with virulence [80]. On the other hand, M1 protein showed the 15I substitution which was detected in all H9N2 viruses, and it is reported to be a mammalian host-specific marker [81]. Genetic analysis of the NS1 protein of the Egyptian isolates showed that all H9N2 viruses had 42Svirulence marker (Table 3). The NS1 protein was 230 aa long and contained the ^227^ESEV^230^PDZ ligand. No deletion was detected at 80–84 in NS1 [82].

Positive selection is a driving force for the increase in the number of genetic variants, allowing the preservation of mutations which favour viral adaptation and survival. Negative selection purifies unfavourable mutations and tends to conserve genes [83, 84]. In the present study, SLAC and MEME were used to analyse the full length genes of for selection pressure [55]. It was found that all H9N2 strains genes reported in the current study were under purifying selection pressure except HA gene, which was found to be under positive selection pressure, where a total of eight positively selected pressure sites for the HA gene were identified as shown in (Table 4). Analysis revealed that two of these positively chosen sites are situated near the receptor-binding locations of the HA protein, specifically at sites (A198 and I235). Receptor-binding preference is crucial for the replication and dissemination of influenza viruses [85]. Amino acid mutations at these locations allow the H9N2 avian influenza virus to adapt to extreme conditions [86]. In the present study, multiple H9N2 virus strains containing mutations related to mammal-adaptation were reported. It was suggested that H9N2 viruses are already able to infect humans and that their capacity to attach to human receptors may be enhanced by continuous evolution and selection on these sits [42, 87] which require further investigations.

## Conclusions

The present study reports the full-length genome sequences of ten AIV H9N2, which were newly identified from Egypt between 2019 and 2023. Phylogenetic analysis revealed that the surface and internal genes which followed the same evolutionary trends and are associated with the H9N2 genotype III Egyptian strains within the H9N2 (G1-H9 4.2). Additional animal model studies are needed to assess the pathogenicity of the current Egyptian H9N2 strains. The implementation and enforcement of strict biosecurity measures are strongly advised to minimize the spread of AIV infections into Egyptian poultry farms. The present study, aimed to investigate the genetic characterization and evolution of avian influenza virus H9N2 in Egypt. The results showed that the H9N2 viruses in Egypt belonged to G1-H9 4.2, and clustered to genotype III of Egyptian strains. All strains carried H191 and 234 L residues in the HA gene, which are mammalian adaptation markers. Many mutations associated with virulence and mammalian infection were detected in internal proteins such as PB2-V504, PB1-F2-N66, PA (V127, L672, and L550), M2-S64, and NS1-42S. With various mammalian and viral substitutions indicating virus evolution, it is recommended that further investigations including functional virological studies are required to monitor the evolution of H9N2 strains and determine the antigenicity of the reported strains which will help understand the potential threats such viruses pose to humans.

## Data Availability

Sequence data generated in the present study which support the findings and conclusions of this study have been deposited in the GenBank, the National Library of Medicine under accession numbers PP345560-PP345569, PP345544-PP345553, PP345534-PP345543, PP345489-PP345498, PP345524-PP345533, PP345513-PP345522, PP345918-PP345927, PP346094-PP346103 for PB2, PB1, PA, HA, NP, NA, MP, and NS segments, respectively.

## Abbreviations

AI: Avian influenza
AIV: Avian influenza virus
HPAI: Highly pathogenic avian influenza
LPAI: Low-pathogenic avian influenza
HA: Haemagglutinin
NA: Neuraminidase
